# Medical applications: a database and characterization of apps in Apple iOS and Android platforms

**DOI:** 10.1186/1756-0500-7-573

**Published:** 2014-08-27

**Authors:** Heather J Seabrook, Julie N Stromer, Cole Shevkenek, Aleem Bharwani, Jill de Grood, William A Ghali

**Affiliations:** Ward of the 21st Century, University of Calgary, GD01 TRW Building, 3280 Hospital Drive NW, Calgary, AB T2N 4Z6 Canada; Department of Medicine, University of Calgary, Foothills Medical Centre, 1403 29th Street NW, Calgary, AB T2N 2 T9 Canada; Department of Community Health Sciences, Institute for Public Health, University of Calgary, Faculty of Medicine, 3rd Floor TRW Building, 3280 Hospital Drive NW, Calgary, AB T2N 4Z6 Canada

**Keywords:** App, Application, Software, Smartphone, Handheld computer, Mobile health

## Abstract

**Background:**

Medical applications (apps) for smart phones and tablet computers are growing in number and are commonly used in healthcare. In this context, there is a need for a diverse community of app users, medical researchers, and app developers to better understand the app landscape.

**Methods:**

In mid-2012, we undertook an environmental scan and classification of the medical app landscape in the two dominant platforms by searching the medical category of the Apple iTunes and Google Play app download sites. We identified target audiences, functions, costs and content themes using app descriptions and captured these data in a database. We only included apps released or updated between October 1, 2011 and May 31, 2012, with a primary “medical” app store categorization, in English, that contained health or medical content. Our sample of Android apps was limited to the most popular apps in the medical category.

**Results:**

Our final sample of Apple iOS (n = 4561) and Android (n = 293) apps illustrate a diverse medical app landscape. The proportion of Apple iOS apps for the public (35%) and for physicians (36%) is similar. Few Apple iOS apps specifically target nurses (3%). Within the Android apps, those targeting the public dominated in our sample (51%). The distribution of app functions is similar in both platforms with reference being the most common function. Most app functions and content themes vary considerably by target audience. Social media apps are more common for patients and the public, while conference apps target physicians.

**Conclusions:**

We characterized existing medical apps and illustrated their diversity in terms of target audience, main functions, cost and healthcare topic. The resulting app database is a resource for app users, app developers and health informatics researchers.

**Electronic supplementary material:**

The online version of this article (doi:10.1186/1756-0500-7-573) contains supplementary material, which is available to authorized users.

## Background

Medical applications (“apps”) for mobile devices such as smartphones and tablet computers provide healthcare professionals, patients, and the public with a growing number of specialized tools and resources. Physicians and medical students have high rates of smartphone and tablet computer ownership [1–3], enabling them to use medical apps. There are over 800,000 apps available for each of the dominant mobile platforms, Apple iOS and Android OS, and a subset of these are categorized by developers as medical apps (2% for Apple iOS [4] and 1% for Android^a^).

A number of researchers have explored the use of smartphones and medical apps amongst medical students, residents and physicians. One survey at a Canadian medical school found that 85% of respondents owned smartphones and most regularly used medical apps [1]. In terms of functionality, medical students tend to use apps that serve as references for disease diagnosis and medications, whereas physicians prefer medical calculators [2]. Similarly, a systematic review of academic literature on healthcare apps identified these app functions, along with literature searching, as the most common [5]. While this same review identified few published studies on apps for patients, all of the apps directed at patients focused on specific health conditions, including chronic illnesses. Reviews of apps that target specific health conditions [6–10] explore a larger number of available apps for patients. These studies provide insight into the range of functions performed by patient apps, such as education and diary/tracking. Taking a somewhat broader view, a review of the 500 most popular medical apps in the Italian Android market identified a range of app purposes, with public education, health diaries and specialized medical calculators being most popular [11]. Similar to the systematic review of the academic literature, these researchers identified more apps for health professionals than for the public. Thus, the existing literature suggests a varied landscape in apps, with different target audiences being offered different kinds of apps. However, to the best of our knowledge, a comprehensive description of the medical app landscape for the dominant platforms has not been developed.

To contribute to the understanding of the broader medical app landscape, we therefore aimed to characterize a representative subset of apps in the medical category of the two dominant platforms: Apple iOS and Android. In doing so, our objectives were to broadly describe the medical app landscape in terms of: 1) target audiences, 2) main functions performed by medical apps, 3) app functions viewed by target audience, 4) cost, and 5) healthcare topics examined by target audience. The database [12] used to catalogue apps is also provided here to support researchers conducting further investigations. This paper shows the diverse and varied landscape of medical apps by presenting data that illustrate the distribution of medical apps across target audiences, app functions, cost and healthcare topics.

## Methods

### Data sources

We selected our sample of mobile apps from the medical category of the two official app stores: iTunes App Store for Apple iOS apps and Google Play for Android apps. At the time of data collection, we were able to access the full range of iTunes medical apps, because iTunes displayed all apps in the medical category, whether free or paid. However, Google’s removal of the full-category view in early 2012 when they rebranded the Android Market as Google Play [13] resulted in only being able to access a subset of Android medical apps in both the free and paid sub-categories. This subset includes the most frequently downloaded apps (i.e., most popular). Apple also removed the full-category view in the Fall of 2012 [14] after our data were collected.

### Identification of app characteristics - database structure

The research team collectively developed a list of medical app characteristics, after examining a random selection of apps. We defined target audience by the role of the group(s) who would use the app as either specified or inferred from the app description (i.e., physicians, medical students, nurses, clinicians - unspecified, other healthcare professionals, patients, the public and others). We added the “clinician - unspecified” target audience after observing that many apps specified a general audience (e.g., “intended for healthcare providers”) or could be used by a range of health professionals. Other healthcare professionals include allied health, pre-hospital care, and alternative medicine. When an app might be used by a person who is under health provider care or managing a long-term condition such as diabetes, we characterized it as a patient app; whereas apps for self-care without clinician over-sight, including home remedies, calorie trackers, home health guides, and medical service locators, were characterized as apps for the public. We identified the function or purpose of an app using the following categories: alternative medicine (e.g., acupuncture, herbal medicine, homeopathy, meditation and yoga), calculator (e.g., pregnancy due date calculators to veterinary medicine dose calculators), conference (e.g., accompanies a conference often providing access to schedules and resources), diagnosis (e.g., ranges from apps for self-diagnosis with heart attack symptoms to psychological tests), education (e.g., educational content usually to support self-directed learning), monitoring/export, motivational, nutrition/diet, and other (e.g., a broad range including tools for people with disabilities, appointment booking and service advertising/locating, patient records/results (e.g., patient management, history taking, access to patient test results), reference (e.g., guides, databases, flash cards, quick references), reminder (e.g., medication reminder, healthcare appointment reminder, and addiction coaching), social media (e.g., support groups for specific conditions), tracking/diary (e.g., to record and/or display measures over time including symptoms, test results, medications taken, weight, fetal growth). We also noted the initial cost of the app in Canadian dollars. Cost does not include in-app purchases or subscriptions required to use an app. We also characterized apps by topics, which includes medical conditions (e.g., diabetes, asthma), areas of study or specialization (e.g., pharmacology, cardiology), and treatments or activities (e.g., surgery, exercise). We developed a database using Microsoft Access 2010 to categorize the apps using the defined characteristics. We included fields for primary through tertiary target audiences and functions, because our preliminary explorations revealed that apps are often targeted at multiple audiences with multiple purposes.

### App characterization

We characterized the apps in both Apple iTunes and Google Play using the developer-written descriptions and, when available, screen captures. Sometimes the app description used the same terms our team had chosen to characterize apps (e.g., physician, tracking). In these cases, we simply recorded the information from the description. More commonly, characterization required interpretation of the app description and screen captures. We also characterized apps thematically by content themes [15]. To identify content themes, we first coded the apps by topics such as medical conditions, areas of study and specializations. Then two researchers reviewed the list of topic codes to develop the higher-level content themes, which were subsequently refined through input from content experts.

To support coding consistency, two researchers independently categorized 50 apps, comparing entries after every ten. Few differences were found, with the exception of topic coding. When discrepancies occurred, they discussed the coding to reach mutual agreement and refined our coding approach. New topic codes were added to a master list as they emerged, providing us with a growing list of keywords to apply to subsequent apps and to use in further analyses.

To assess congruence between the developers’ descriptions and the actual app, we installed a random selection of 20 Apple iOS apps from the first 600 apps we had catalogued. We characterized the installed apps by working with the apps and then compared this characterization to that derived from the developers’ descriptions. Coding was consistent using the installed app and developer description except in the case of one paid app with a poorly written (possibly auto-translated) description. We performed a similar verification process with a random selection of 10 popular Android apps from the first 60 apps we had catalogued. Again, coding was consistent, assuring us that the app store description could be used to characterize the apps.

### Inclusion and exclusion criteria

A number of filtering processes were applied to the data at the time of collection and during refinement to ensure data quality.

#### Medical category

All apps were sampled from the medical category of the app markets. We included apps with the primary category listed as “medical” in Apple iTunes. Apps that did not have medical as a primary category (e.g., health and fitness, lifestyle) were excluded (approximately 25%). Google Play allowed developers to choose only one category for their app; therefore, manual exclusion of apps based on category was not necessary.

#### Medical apps

Available apps from the medical category were included. We excluded apps that did not feature health or medical content, such as music apps. Some apps appeared to be erroneously categorized.

#### Date released or last updated

We limited our sample to apps released or updated between October 1, 2011 and May 31, 2012. Active apps are often updated to add functionality, fix software problems, and show the app is current. We selected the date range based on a random selection of Apple iOS medical apps (220 of approximately 7000), for which the distribution with respect to release/update date fell off significantly prior to October 2011.

#### English language

We excluded apps that used a language other than English as the primary language.

### Analysis

We designed queries in Microsoft Access to explore subsets of the database. We extracted data by overall target audience, function, cost and topic, and also explored the latter three by their target audiences. We summarized data coded in the primary and secondary categories of the database for target audience and function, choosing these to capture the dominant categories for our analysis rather than a more exhaustive list. We used Microsoft Excel 2010 to calculate descriptive statistics and generate the figures included in this report.

## Results

### Number of apps in our sample

We retained 4857 medical apps in our final sample: 4561 Apple iOS and 293 Android. The final Android sample is small compared to our Apple iOS sample and is limited to apps that were most frequently downloaded because of the app search site changes as described above. Due to the differences in the samples, we present the results for the two platforms separately.

### Characterization of Apple iOS apps from the medical category

#### Distribution of Apple iOS apps by target audience

**Figure 1:**
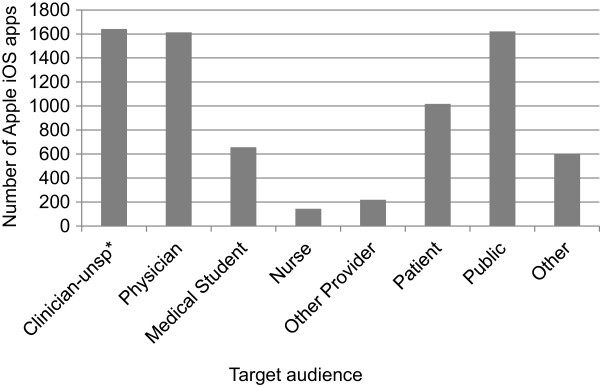
**Distribution of Apple iOS medical apps by target audience.** The clinician - unspecified category includes physicians, nurses, medical students and pharmacists and was applied when multiple audiences were possible or a general description such as “health provider” was supplied in the description. (unsp* = unspecified).

Figure 1 illustrates the number of medical apps identified for each target audience in our Apple iOS sample. Totals include the primary and secondary target audience fields in the database. The proportion of Apple iOS medical apps intended for public use (35%) is similar to the proportion of apps for physicians (36%). “Other” includes apps for researchers and for administrators. Figure 1 illustrates that relatively few Apple iOS apps were meant for nurses (3%) and other healthcare providers (e.g., physiotherapists and dieticians) (5%). As an extension of these data, we found that few apps were intended for use by both patients and providers (physicians or clinicians) (0.8%).

#### Distribution of Apple iOS apps by function

**Figure 2:**
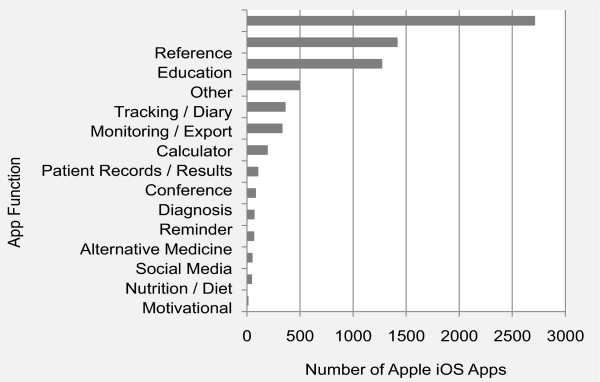
**Distribution of Apple iOS apps by function.**

**Figure 3:**
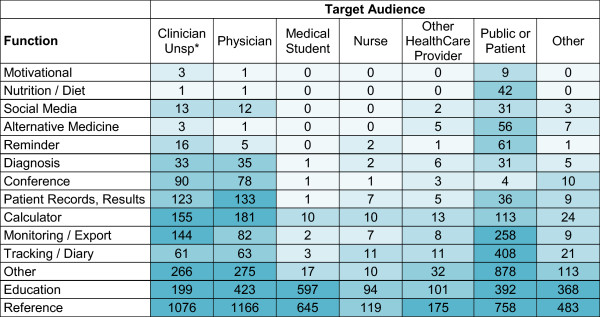
**Colour-coded concentration graph of apps by function and target audience.** Data are coloured by quintile. The colour intensifies as the number of apps in the quintile increases. (unsp* = unspecified).

Figure 2 shows app distribution by function or purpose in our Apple iOS sample. Reference and education apps dominate. The third largest functional category is “other,” which includes apps for clinic advertisements and accounting purposes. Apps that perform tracking and monitoring functions are common, as are calculators.Figure 3 displays a colour-coded concentration graph of apps by target audience and function. Data are coloured by quintile; the colour intensifies as the number of apps in the quintile increases. Figure 3 shows that reference apps are the most common function for all audiences. Other functions have more varied target audiences. Figure 3 also illustrates that apps that remind, monitor and track are often intended for patients or the general public, as are social media apps; whereas, calculator, reference and conference apps are more often intended for physicians.

#### Distribution of Apple iOS apps by cost

Free apps dominate the Apple iOS medical category and most of the paid apps cost $5CDN or less to install. Few apps cost more than $20.00 to install. The average cost of paid Apple iOS medical apps is $11.26. By comparison, average costs for some specific audiences are as follows: physician $18.72, nurse $10.21 and patient $8.30. The modal cost among non-free apps is $0.99.

#### Distribution of Apple iOS apps by content themes

Our analysis of the coded topics resulted in 29 themes (see Additional file 1). Additional file 1 illustrates that the total number of apps varies by content theme and by target audience within the themes. For example, anaesthesiology and radiology contain relatively large numbers of apps for physicians. Conversely, a large proportion of pain and migraine management apps target both patients and the public; while diet apps mainly target the general public with few apps for patients and none for health professionals.

### Characterization of popular Android apps from the medical category

#### Distribution of popular Android apps by target audience

**Figure 4:**
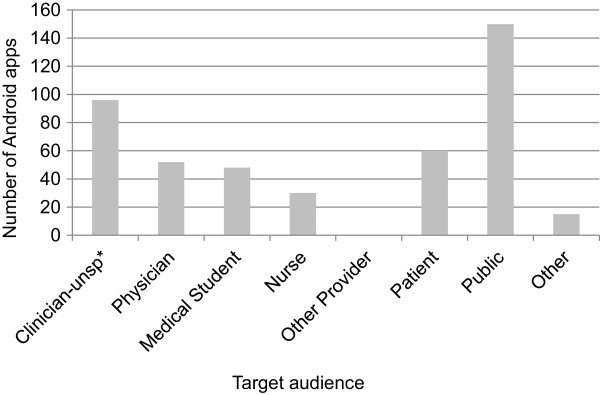
**Distribution of popular Android medical apps by target audience.** The clinician - unspecified category includes physicians, nurses, medical students and pharmacists and was applied when multiple audiences were possible or a general description such as “health provider” was supplied in the description. (unsp* = unspecified).

Figure 4 illustrates the number of medical apps identified for each target audience in our sample popular Android medical apps. These apps are dominated by apps intended for the public (51%). Although there were many apps targeting nurses, there were 42% more apps coded for physicians.

#### Distribution of popular Android apps by function

**Figure 5:**
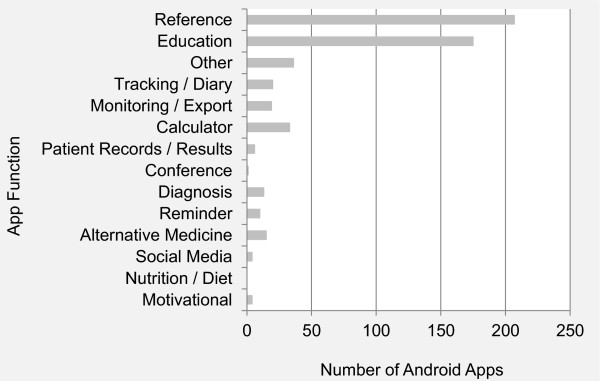
**Distribution of popular Android medical apps by function.**

Figure 5 shows app distribution by function or purpose in our Android sample. Reference and education apps are the largest functional category followed by “other,” which includes apps for clinic advertisements. Apps that perform tracking and monitoring functions are common, as are calculators.

#### Distribution of popular Android apps by cost

Over 75% of the most popular apps in the Android medical category are free to install. Most popular paid Android apps cost between $2 CDN and $5 CDN. The average cost of popular paid Android medical apps is $8.04. By comparison, the average costs for some specific audiences are as follows: physician $12.74, nurse $5.27, and patient $1.97.

## Discussion

This study catalogued a large number of apps from the medical category to describe the medical app landscape in the two dominant platforms: Apple iOS and Android. In doing so, we identified target audiences, functions, costs and content themes. Overall, our results reveal the diversity of the medical app landscape. Our colour-coded “concentration graph” illustrates the distribution of apps across target audiences and functions. In addition, we describe a method for conducting research in this dynamic domain and present the database developed for the study with the data in an open-access format so that researchers and app developers can explore further questions [12].

The recent explosion of medical apps has attracted the attention of a wide audience, including app developers, clinicians, the general public, regulators and the popular media. To date, in the medical informatics literature, the medical app landscape has not been systematically described. The present study updates and extends the existing body of literature by presenting a cross-sectional categorization of apps that represents the scope of the medical app landscape in the two dominant platforms at the time of study. Previous studies provided insights into the functions, target audiences and quality of specific subsets of the medical app landscape; these studies include apps for specific conditions, such as diabetes [6, 16], asthma [7], colorectal disease [9] and melanoma [10], as well as medical specializations [17]. Our study reviews the broad landscape, investigating the number of apps for various functions and healthcare content themes. In doing so, our results reveal an abundance of apps, which vary in number by functions and content themes, and identify numerous additional subsets of the medical app landscape in need of further investigation into quality.

Our data suggest changes to overall app development trends relative to previous studies and also reveal new levels of detail in app distribution that provide a starting point to investigate future trends. Previous studies indicate that apps for health professionals were most prevalent [5, 11]. However, we found a balance between apps for providers and the public in the Apple iOS platform. Our results reveal a large number of apps available to physicians; however, physicians actually report using few apps and typically use general-purpose medical apps such as calculators and drug references [2, 17, 18]. There is a clear dominance of apps for the public in our sample of popular Android apps. Our results align with trends in device ownership: physicians prefer Apple devices [18], whereas Android devices are more common overall [19]. Further, our classification distinguished between patients and the public, with apps for the public directed towards self-care. Few apps for patients would also be used by providers, suggesting little information sharing or communication. Our results suggest a smaller presence for clinicians in the medical app landscape than previously described. Our data collection method also allowed us to specify clinicians as a general target audience as well as physicians, medical students, nurses and “other health professionals” as more specific audiences. In doing so, we provide a unique view of the app landscape that highlights the dearth of apps for nurses and other health professionals. Thus our results temper the call to curb the proliferation of medical apps [20] by highlighting areas where there are few apps, while recognizing the need to ensure these apps provide quality tools and resources [17]. Interestingly, though there is a large variety of apps available, downloads are generally confined to the most popular apps [11]. It is worth noting that neither general availability nor number of downloads tells us about initial or ongoing app-use. The “who, what and why” of app-use remains an important area for future research. It is also important to note that while our data reveal that most medical apps are free, the initial cost of apps does not reflect the total cost to use an app, with an increasing amount of app income coming from in-app purchases [21].

We also describe a method that can be used by future researchers to conduct similar studies, whether focusing on medical apps or exploring other app market categories. Researchers conducting app reviews face considerable challenges, including lack of an established method, descriptions that lack a consistent format or metadata, and volatility of app store presentations. Had we devised a means of extracting all required data from the app markets at the outset of the study, the risks of volatility would have been mitigated. This should be taken into consideration for those conducting similar studies in the future.

The work presented here is not without limitations. The scope of this review was limited to English apps in the Medical category, excluding potentially rich data within the Health and Fitness and Education categories and in other languages. These exclusions were mindfully applied to limit the scope to apps we could reliably characterize and to focus on apps developers defined as medical. A second limitation of this work is that our sample of Android apps was limited to only the most popular apps. Popularity is based on the number of downloads; consequently, the sample may be biased towards free and inexpensive apps, exclude specialized clinical apps and overemphasize patient/public apps. By extracting data from the two dominant platforms, we were able to make high-level comparisons and observe that the general trends in target audience, function and cost are similar, despite the potential bias. Our small Android sample did not allow us to analyze these data across target audience and functions, and target audience and themes. Finally, the cataloguing process depended on our ability to interpret app store descriptions. Deciding how to characterize an app in terms of health professionals and key functions often required interpretation. We iteratively compared and refined our cataloguing to achieve consistency; however, the subjective nature of coding remains in any study of this kind. Guidelines for medical-app descriptions that include target audience, functions and keywords would mitigate this challenge and also benefit those attempting to find apps.

## Conclusions

In this study we used an approach analogous to a scoping review of the literature to assess the “size and scope… nature and extent” [22] of the medical app landscape of the two dominant platforms: Apple iOS and Android. We describe the domain in terms of target audience, app function, cost and content themes. The resulting snapshot of the medical app landscape may benefit medical app users, developers, and future researchers wishing to catalogue mobile apps. For medical app users, the information here could help them search for and identify apps. Similarly, the scoping review provided by the present study could support efforts to manage app proliferation and improve app discoverability. For developers, our data may expose key insights into areas of future app development opportunities. Finally, for future researchers, this time-stamped data collection can be used in a retrospective comparison and as a guide for conducting similar reviews.

### Availability of supporting data

The data set supporting the results of this article is available in the Labarchives repository, DOI:10.6070/H4QV3JGM.

## Endnote

^a^Calculated using data available from appbrain.com retrieved on Feb 10, 2013. The total number of apps in the Android Medical category is available at http://www.appbrain.com/stats/android-market-app-categories and the total number of Android apps is available at http://www.appbrain.com/stats/number-of-android-apps.

## Electronic supplementary material

Additional file 1: **Distribution of Apple iOS apps by themes and topics for each target audience.** (PDF 62 KB)
