# Supplementary material for: Medical applications: a database and characterization of apps in Apple iOS and Android platforms
Source: BMC Res Notes. 2014 Aug 27;7:573. doi: 10.1186/1756-0500-7-573 (PMC4156621; doi:10.1186/1756-0500-7-573)
Supplement: Supplementary file 1 — Additional file 1: Distribution of Apple iOS apps by themes and topics for each target audience. (PDF 62 KB) [file 13104_2014_3107_MOESM1_ESM.pdf]

Additional file 1. Distribution of Apple iOS apps by themes and topics for each target audience.

Theme	Topics	Total # of Apps*	Physician	Medical Student	Nurse	Other HCP	Patient	Public	Other
Alternative Therapies	acupuncture, alternative medicine, chiropractic, herbal medicine, reflexology, relaxation	85	5	0	0	0	40	47	7
Anatomy	anatomy	346	124	292	9	12	3	36	104
Anesthesiology	anesthesiology	45	34	7	1	0	3	1	3
Bones and Joints	arthritis, orthopedic, podiatric, rheumatology	57	42	5	1	2	5	3	5
Cardiology and Vascular Health	cardiology, heart disease, hypertension, stroke	196	125	20	8	1	40	30	12
Dental	dental	227	44	14	1	12	142	147	11
Dermatology and Cosmetic Procedures	cosmetic procedures, dermatology	43	22	5	0	1	11	15	2
Diet	diet, nutrition, obesity, weight	88	0	0	0	0	5	86	0
Exercise	exercise	24	0	0	0	2	5	19	1
First Aid and Emergency Medicine	emergency, EMS, first aid, fever	51	4	0	0	6	1	41	3
Hearing	audiology, hearing	27	4	1	3	2	12	10	2
Maternal and Baby Health, and Parenting	baby, baby nursing, child, childbirth, parenting, pediatrics, pregnancy	258	37	4	4	2	8	207	9

Theme	Topics	Total # of Apps*	Physician	Medical Student	Nurse	Other HCP	Patient	Public	Other
Mental Health and Wellness	alzheimer's, autism, depression, eating disorder, learning disability, mental health, psychiatry, psychology, stress)	85	12	3	0	4	37	44	14
Neurology	neurology	61	36	14	0	2	6	6	11
Oncology and Cancer Care	cancer, chemotherapy, oncology	99	50	7	0	0	25	25	5
Pain and Migraine Management	migraine, pain	53	4	1	0	4	41	24	1
Pathology	pathology	14	9	3	1	0	1	0	1
Pharmacy and Pharmaceuticals	antibiotics, drugs, medication, pharmacology, pharmacy, vaccination	188	51	9	8	30	64	74	14
Radiology	radiology, ultrasound, sonography	93	63	19	1	3	2	1	19
Rehabilitation	physiotherapy, rehabilitation	5	0	0	0	3	1	0	0
Respiratory Health	asthma, pneumonia, pulmonology, respiratory	19	5	3	0	0	9	2	2
Surgery	arthoscopy, endoscopy, plastic surgery, surgery, transplantation, urology	150	63	15	4	66	62	58	14
Veterinary Medicine and Animals	veterinary	78	22	0	0	0	1	30	26

Theme	Topics	Total # of Apps*	Physician	Medical Student	Nurse	Other HCP	Patient	Public	Other
Vision	impaired vision, ophthalmology, optometry	190	61	8	2	3	100	103	3
Women's Health	birth control, gynecology, menstruation	30	6	7	1	0	3	16	3
Broader Medical Topics	general, treatment	1238	63	143	55	58	185	179	50
Miscellaneous Medical	disability, speech, sleeping, game	75	4	2	0	1	28	46	2
Non-Clinical Medical Apps	communication, donation, financial, other, patient records	560	213	42	6	24	69	190	103
Other Internal Medicine	allergies, diabetes, endocrinology, hematology, hepatitis, HIV, AIDS, nephrology, otolaryngology, phlebotomy, smoking	127	44	10	13	0	64	15	8
Total		4512	1147	634	118	238	973	1455	435

*The total number of apps in a theme is the actual number of apps. If an app in that theme was assigned more than target audience, it was only counted once. Therefore the total number of apps is seldom the same as the total for all target audiences.
